# A human in the loop approach to applying large language models for farm management insight

**DOI:** 10.1038/s41598-025-30991-6

**Published:** 2025-12-02

**Authors:** Spyridon Mourtzinis, Tatiane Severo Silva, Jason Chor Ming Lo, Damon L. Smith, Shawn P. Conley

**Affiliations:** 1 Department of Plant and Agroecosystem Sciences, University of Wisconsin- Madison, 1575 Linden Dr, Madison, WI 53706 USA; 2 Data Science Institute, University of Wisconsin-Madison, 447 Lorch Ct, Madison, WI 53706 USA; 3 Department of Plant Pathology, University of Wisconsin-Madison, 1630 Linden Dr, Madison, WI 53706 USA

**Keywords:** Environmental sciences, Plant sciences

## Abstract

Sustainable food production and security depend on increasing agricultural productivity within existing arable land. This necessitates the effective translation of complex agronomic research into actionable, field-specific crop management recommendations. Despite substantial advances in agricultural research, a persistent knowledge-practice gap continues to impede the widespread adoption of evidence-based management practices. We evaluate whether large language models (LLMs) can bridge this gap by generating crop management recommendations from scientific literature. Using US soybean production as a case study, we developed a semi-automated, human-in-the-loop pipeline (hereafter called “our system”) adhering to systematic review protocols. Our system demonstrated high accuracy for literature screening, outperforming standalone models. However, when generating a general soybean management plan, expert evaluations rated two commercial LLMs’ output more favorably than the plan from our system. This work highlights the need to develop systems that address user trust and provide tailored, field-specific advice that is both trustworthy and practically useful for farming communities.

**Supplementary Information:**

The online version contains supplementary material available at 10.1038/s41598-025-30991-6.

## Introduction

Global population growth, along with rising incomes in developing countries, is expected to exert significant pressure on food production systems worldwide^1^. Achieving sustainable increases in crop yields will require intensifying production on existing cropland while minimizing ecosystem degradation and greenhouse gas emissions^2^. Agricultural research plays a crucial role in guiding farmers toward sustainable, productivity-enhancing practices. However, well-documented challenges—such as high environmental variability, fragmented datasets, complex scientific jargon, and limitations in extrapolating findings—hinder the translation of research into actionable recommendations^3^. Furthermore, the rapid growth in agricultural research output coupled with reductions in Extension and outreach staff exacerbates the challenge of distilling practical insights to support on-farm decision-making.

Systematic reviews offer a transparent and rigorous methodology for synthesizing evidence to answer a focused research question^4^. The process involves defining research questions, developing a search strategy, screening studies against predefined criteria, and extracting and synthesizing data^5^. The outcomes can help bridge the gap between fragmented information and comprehensive, actionable insights. However, producing such reviews remains a time- and resource-intensive process, leaving a persistent gap between published scientific findings and the practical advice reaching farmers.

Recently, the field of artificial intelligence has witnessed rapid advancements in the development of large language models (LLMs)^6–8^. These models have demonstrated capabilities in answering agriculture-related questions^9^, generating pest management recommendations^10^, and reviewing scientific publications^11^. These advancements suggest that LLMs could automate time-consuming parts of the knowledge synthesis process. However, current LLM-based tools face challenges, including a lack of control over the knowledge extraction process and the risk of “hallucinations” - where models generate inaccurate or false information^12^. Such fabrications can negatively affect downstream decision-making, challenging user trust and the adoption of generative AI systems.

Here, we explore the use of LLMs to support soybean management planning in the US and evaluate the quality of the generated recommendations through expert review. We focus on US soybean (*Glycine max* (L) Merr.) production, which accounts for approximately 30% of global production^13^, as proof of concept. Unlike traditional reviews, we leverage LLMs in a human-in-the-loop workflow to enable a scalable synthesis of agricultural knowledge. Our system involves a semi-automated, human-in-the-loop pipeline adhering to systematic review protocols for literature screening, distilling, and summarizing information and generating a general soybean management plan. Our approach focuses on extracting actionable insights that directly inform farm management decisions, laying the groundwork for efficient, continuously updated (“living”) literature reviews in agriculture.

## Methods

**Fig. 1:**
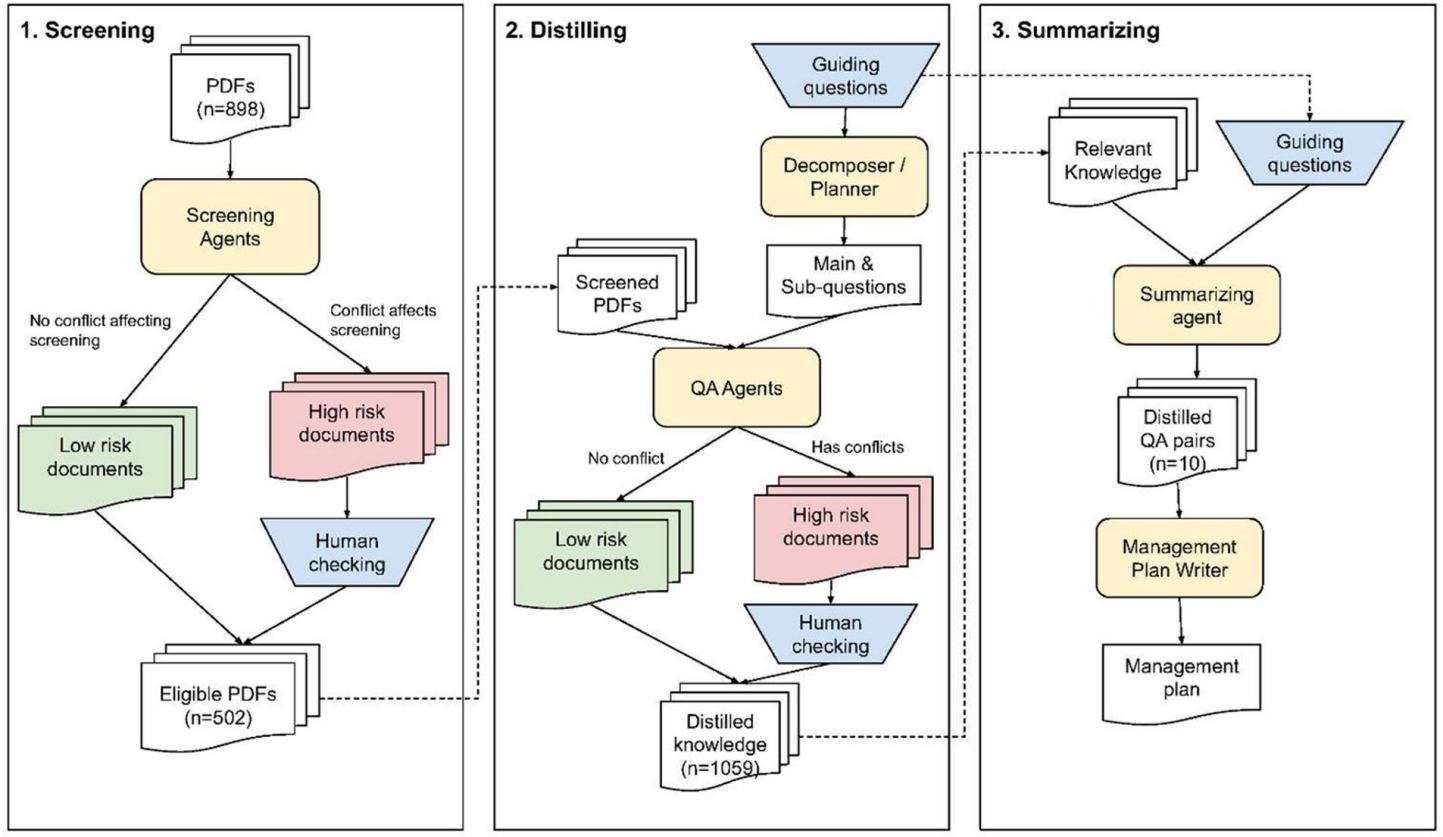
Workflow description from screening, distilling and summarizing data from research studies. The screening process selects PDFs that meet the inclusion criteria. The distilling process extracts relevant knowledge for each question-PDF pair. The summarizing step generates an expert-guided, fully traceable knowledge summary for soybean farm management planning. Large language model (LLM) tasks are shown in yellow, and human tasks are shown in blue.

We conducted a systematic literature search following established meta-analysis protocols to identify studies relevant to soybean management practices in the US. The workflow we followed is shown in Fig. 1. The search strategy employed the PICO framework (Population, Intervention, Comparator, Outcome), using three key components: Population (soybeans), Intervention (management practices), and Outcome (yield responses).

The search was executed in the Web of Science Core Collection database using the field tag TS (Title, Abstract, Keywords) with publication years restricted to 2015–2025 to capture contemporary agricultural management practices. Search terms were iteratively refined through testing various keyword combinations against known relevant articles to optimize retrieval accuracy (Table S1). Database filters were applied to include only English-language publications and studies conducted within the US. The initial search yielded 990 articles, which were exported in Excel format and accessed via DOI links. Manual screening excluded 92 articles that were not conducted in the US or did not focus on soybean as the primary crop of interest, resulting in a final dataset of 898 articles. All retrieved PDFs were converted to Markdown format using the PyMuPDF Python package to facilitate subsequent automated processing.

A multi-model large language model (LLM) screening approach was implemented to evaluate full-text articles against predefined inclusion criteria. Four distinct LLMs (OpenAI-GPT-4.1-mini-2025-04-14, Gemini-2.5-flash-preview-04-17, Deepseek-r1-70b, Llama3.3–70b) independently assessed each study for the following criteria: (1) Geographic scope: Conducted within the US, (2) Crop focus: soybean, (3) Data availability: Includes quantitative yield measurements, 4) Experimental setting: Field-based studies (studies performed solely in greenhouse were excluded), 5) Study design: Primary research (meta-analyses and reviews excluded), and 6) Research context: Field experimental conditions. These models were selected through small-scale testing that balanced performance with available computational resources.

For each criterion, LLM consensus was required for study inclusion. In cases where LLM assessments resulted in tied decisions affecting downstream inclusion, review by our team (hereafter called “experts”) was conducted to resolve discrepancies and ensure accurate study selection. Additionally, a random sample comprising 20% of the screened articles was independently evaluated by human experts to establish ground truth data. This validation dataset was used to assess: (1) individual LLM performance accuracy, and (2) overall human-in-the-loop pipeline effectiveness.

Experts developed ten key research questions addressing critical farm management decisions (Table 1). The questions were intentionally framed to reflect how a practitioner (e.g., a farmer or agronomist), rather than a research scientist, might seek information. Given the complexity of these questions, each was systematically decomposed into specific sub-questions using in-context learning with LLMs, guided by two human-provided examples of question decomposition. For example, the question “How do different seed treatments (insecticide or fungicide) impact soybean yield when planted before May 1 compared to after May 1?” was decomposed into: Were insecticide or fungicide seed treatments evaluated in the study? Did the study compare planting timing before versus after May 1? Was soybean yield measured as a primary outcome variable? For each question-paper combination, multiple LLMs independently extracted relevant information, including: (1) determination of study relevance to the specific question, (2) supporting textual evidence from the publication, and (3) synthesized answers based on reported findings. The prompts and output format is shown in Tables S2 and S3.

**Table 1:** Guiding questions used for screening the synthesized data corpus (see Fig. 1).

1	How do different seed treatments (insecticide or fungicide) impact soybean yield when planted before May 1 compared to after May 1?
2	What is the effectiveness of foliar fungicide applications in controlling white mold and improving soybean yield in fields where white mold is a primary concern?
3	How does the application of foliar fungicide at the R3 growth stage for general disease control affect soybean yield compared to other growth stages?
4	Which row-spacing and seeding rate combinations are most effective in maximizing soybean yield in the North Central U.S. and how do these compare to the mid-south and southern U.S.?
5	What environmental or planting date conditions best support the effectiveness of insecticidal or fungicide seed treatments in protecting soybean yield?
6	How do no-till practices influence insect or slug pest pressures or soybean yield in different regions?
7	What are the primary factors that predict foliar or root diseases in soybeans across various U.S. regions and how do these factors interact with pest management practices?
8	How does the presence of cover crops affect weed emergence, insect pest pressures, and overall soybean yield?
9	What are the long-term trends in soybean yield improvement associated with changes in planting dates, row spacing, tillage practices, or other agronomic inputs?
10	Which pest management strategies (e.g., seed treatments, scouting-based treatments) are most consistent in protecting soybean yield under varying environmental or biotic conditions?

A consensus approach was employed whereby papers were considered relevant to a specific question only when at least two LLMs independently identified relevant data. Due to performance issues—specifically, a higher frequency of incorrect or irrelevant answers—we limited this step to the two top-performing models (OpenAI and Gemini). For papers meeting this threshold, extracted answers were processed through a final inconsistency detection algorithm to identify and resolve conflicting interpretations. Human expert review was conducted for all cases where inconsistencies were detected between LLM extractions, ensuring accuracy and reliability of the final synthesized recommendations.

Finally, we evaluated the ability of our system and four other AI platforms on June 3, 2025 (OpenAI Deep Research with 4o, Gemini Deep Research with 2.5 Pro, Perplexity Research, Crop Wizard v1.5) to develop a general US-wide soybean farm management plan. The first three are agentic AI systems designed for deep research tasks, capable of autonomously planning, retrieving, and synthesizing information to answer complex questions. In contrast, Crop Wizard is a retrieval-augmented generation (RAG) system tailored specifically for agricultural use cases. Each system was given the prompt: “Create a soybean farm management plan tailored to U.S. conditions.”. The resulting plans were evaluated through a survey completed by 44 independent soybean experts from the U.S., including university faculty, state soybean Extension specialists, and representatives from state or national soybean boards. Experts rated the correctness, completeness, practicality, and relevance of each plan and provided an overall rating. The survey also collected data on expert sentiment regarding the use of AI in agriculture.

## Results

The four LLMs demonstrated varied performance in screening research articles against inclusion criteria (Table 2). The commercial models (OpenAI and Gemini) generally outperformed the open-source models (Llama and DeepSeek), achieving F1 scores ranging from 0.81 to 0.99 across the criteria. The open-source models had a wider performance range, with F1 scores between 0.43 and 0.92. The human-in-the-loop approach, which used expert review to resolve ties, consistently achieved the highest F1 scores, reaching near-perfect or perfect scores (0.98 to 1.00) for all criteria. The most challenging criterion for all individual LLMs was identifying studies that were not conducted in a greenhouse, where F1 scores were as low as 0.43 (Llama) and 0.48 (DeepSeek). The human-in-the-loop method successfully resolved these ambiguities, achieving an F1 score of 1.00 for this criterion and an overall combined screening F1 score of 0.96, surpassing the best individual model (OpenAI, 0.90).

**Table 2:** Comparison of F1 Scores for Human-in-the-Loop and LLM approaches in screening research articles. The F1 score (ranging from 0 to 1) measures a model’s accuracy by calculating the harmonic mean of its precision and recall. Precision is the proportion of positive identifications that were actually correct, while recall is the proportion of actual positives that were correctly identified. The F1 score is particularly useful for classification tasks like literature screening because it provides a more robust measure than simple accuracy, especially when classes are imbalanced (e.g., a large number of irrelevant articles). A higher F1 score indicates better classification performance.

Screening criteria	Llama	DeepSeek	OpenAI	Gemini	Human-in-the-loop
Is primary study	0.82	0.82	0.98	0.99	0.99
Soybean-focused	0.92	0.91	0.97	0.97	0.99
Yield data collected	0.82	0.80	0.92	0.94	0.98
Conducted in the US	0.84	0.92	0.99	0.99	1.00
Is field study	0.92	0.92	0.94	0.97	0.98
Not conducted in greenhouse	0.43	0.48	0.81	0.81	1.00
Combined Screening Results	0.71	0.70	0.90	0.89	0.96

The human-in-the-loop method uses a majority vote from the four LLMs, with a human expert serving as the tiebreaker.

**Fig. 2:**
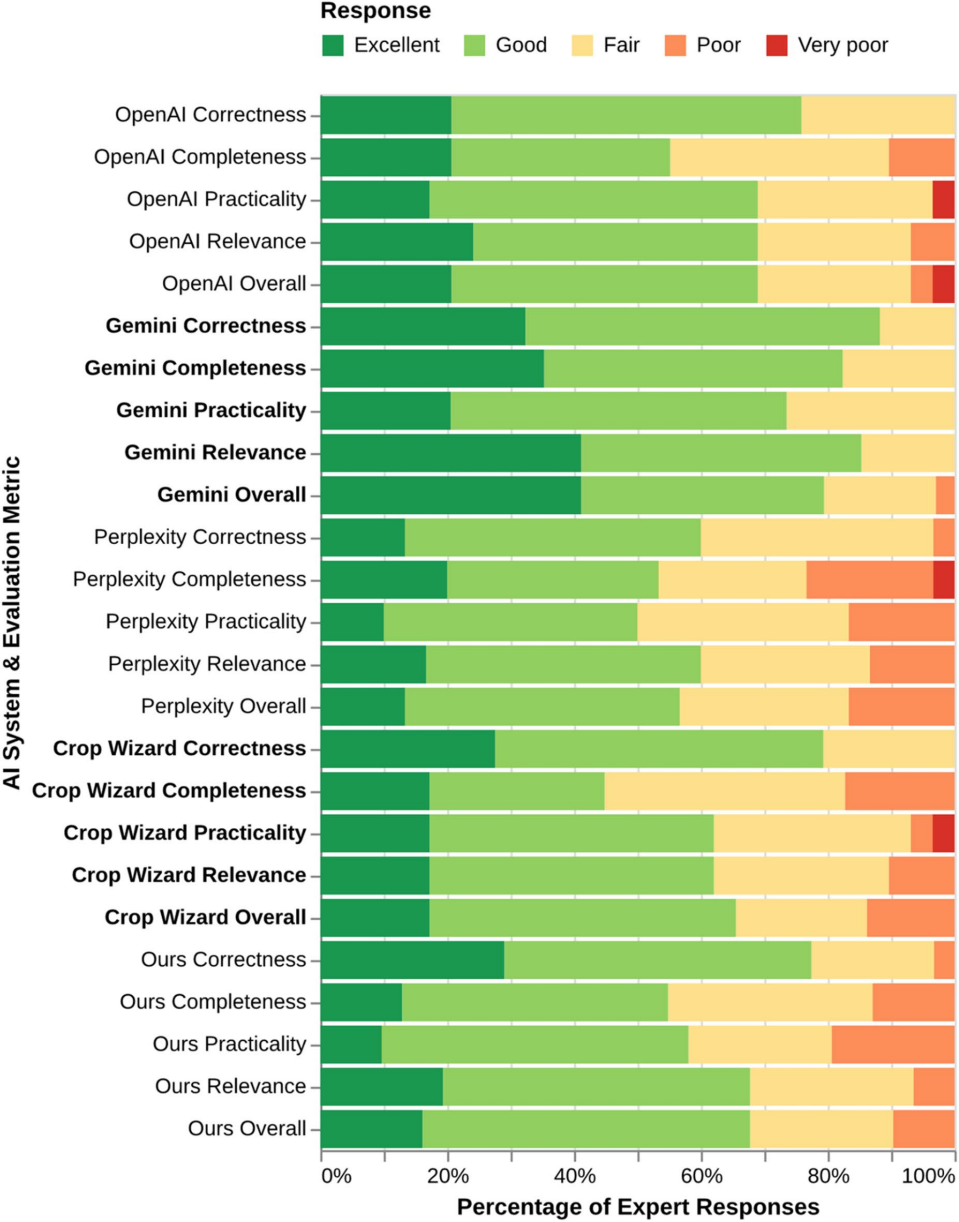
Evaluation of soybean management plans tailored to US conditions generated by OpenAI, Gemini, Perplexity, Crop Wizard and our system. Management plans were evaluated by 44 soybean research experts across the north central US via survey questionnaire.

The soybean management plans generated by the five AI systems were rated differently by the 44 domain experts (Fig. 2). Based on the expert evaluations, Gemini’s plan received the highest overall ratings, with approximately 79% of experts rating it as ‘Good’ or ‘Excellent’. The OpenAI plan followed with about 69% positive ratings, and our system’s plan was rated ‘Good’ or ‘Excellent’ by approximately 68% of experts. The plan from Perplexity received the lowest overall positive rating at 57%, while Crop Wizard received an overall rating of 66%.

Specifically, for ‘Correctness’, Gemini’s plan was again the top performer, with 88% of experts giving it a ‘Good’ or ‘Excellent’ rating. Our system’s plan was also rated highly for correctness, receiving a combined ‘Good’ or ‘Excellent’ rating from 77% of experts, just ahead of OpenAI’s plan (76%). For ‘Completeness’, Gemini’s plan was rated highest, with about 82% of experts rating it ‘Good’ or ‘Excellent’. Our system’s plan received a lower positive rating for completeness (55%). Regarding ‘Practicality’, Gemini’s plan was also perceived most favorably, with over 74% of experts giving it ‘Good’ or ‘Excellent’ ratings. In contrast, our system’s plan received the highest percentage of ‘Poor’ ratings for practicality, at nearly 19%.

No system was entirely free of negative ratings, as every system received at least one ‘Poor’ or ‘Very poor’ rating on some metric. These negative evaluations can likely be attributed to LLM errors or the omission of key aspects of soybean management. It is critical to note that the management plans were generated from a prompt requesting general recommendations applicable across the US, rather than for specific agroecological regions. Consequently, the omission of region-specific details, which are critically important to agricultural experts, likely contributed to the negative ratings. For example, an optimal planting date or pest management strategy for soybeans in the north-central US would differ significantly from best practices in the eastern US. Therefore, a generalized recommendation, while factually correct on a broad scale, could be rated impractical or incomplete by an expert evaluating it for their specific locale.

**Fig. 3:**
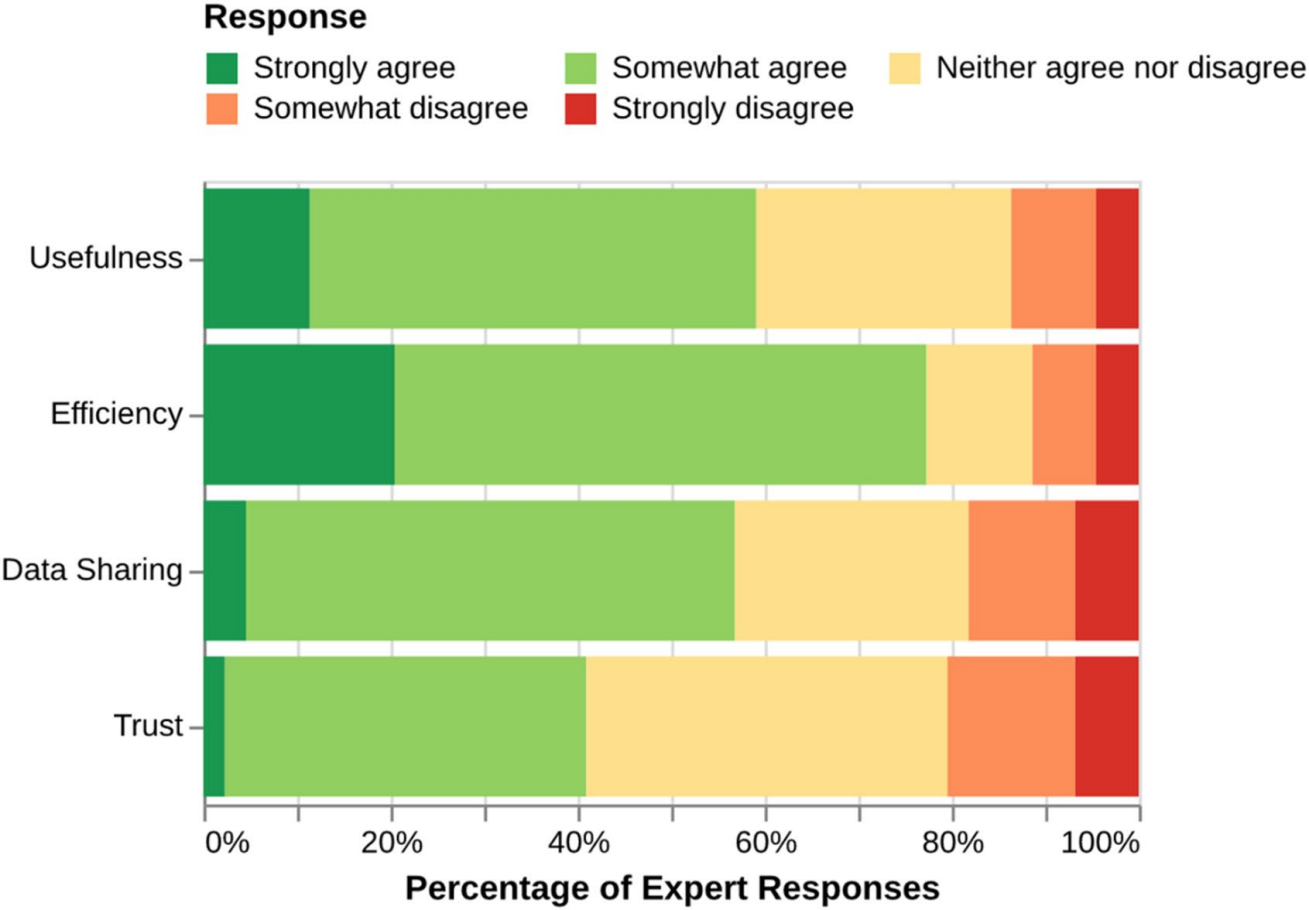
Sentiment of agricultural experts towards the application of artificial intelligence in farming, based on survey responses.

The survey of agricultural experts revealed a generally positive but nuanced sentiment toward the application of AI in their field (Fig. 3). There was strong agreement on the potential benefits for increased efficiency, with approximately 77% of respondents selecting ‘Strongly agree’ or ‘Somewhat agree’. Sentiment regarding increased usefulness was also positive, with about 59% of experts agreeing. However, sentiment regarding trust in AI was more divided: only about 41% of experts agreed that AI is trustworthy, while a similar proportion (39%) remained neutral (‘Neither agree nor disagree’), and 20% expressed some level of distrust. Opinions on data sharing for AI development were also mixed. While a majority (about 57%) agreed to share data, approximately 25% were neutral, and nearly 18% were hesitant, expressing disagreement. This suggests that while experts recognize the potential utility of AI, building trust and addressing data-sharing concerns are critical for its broader adoption.

## Discussion

This study demonstrates the potential and current limitations of using LLMs to bridge the gap between scientific research and practical farm management. Our results from the initial literature screening process show that a human-in-the-loop approach is currently essential for achieving high accuracy, significantly outperforming the best standalone model. This greater performance was most evident in nuanced tasks, such as differentiating between field and greenhouse studies.

This finding, while specific to LLMs, aligns with a foundational principle in the broader field of agricultural AI. Our system, which involves expert intervention to validate and correct outputs, is a modern application of the same core principle that makes ‘supervised’ or ‘guided’ machine learning so effective: human expertise is indispensable. While traditional supervised learning embeds this expertise upfront by training models on human-labeled data^14–16^, our human-in-the-loop approach applies it as an iterative validation step.

Both methods confirm that human guidance is critical for avoiding the incorrect or misleading interpretations that can arise from purely unsupervised approaches^17^. This highlights that while LLMs can greatly accelerate the initial stages of a systematic review, as seen in other fields^18^, this human expertise remains indispensable for ensuring the quality and accuracy of the selected evidence base^19^.

In the generation of farm management plans, the plan generated by the standalone Gemini model received the most favorable ratings from experts, particularly for correctness and completeness. In contrast, our system, despite being built on a more rigorously screened dataset, received lower ratings for completeness and practicality. This finding suggests that the data corpus we synthesized was not large and diverse enough to capture all specific aspects of soybean management (e.g. crop marketing). Additionally, the final synthesis and presentation of information are as important as the quality of the underlying data. Furthermore, the practicality of the generated plan is inherently limited by the nature of its source material: peer-reviewed research papers. These studies are typically conducted in researcher-managed experimental stations under conditions that may not fully represent the spatiotemporal variability and operational complexities of commercial farms. Our system’s lower practicality ratings may, in part, reflect this disconnect. While synthesizing formal research is a crucial first step, the future of LLM-powered farm management tools must involve the integration of diverse, large-scale data sources, such as commercial performance data from on-farm trials and precision agriculture technologies.

Generating a single plan for the entire US likely omitted the crucial region-specific details that agricultural experts rely on for making practical, on-the-ground decisions. This underscores a key challenge for developing effective agricultural AI: the need to move beyond generalized knowledge to location-specific^3^, actionable intelligence with some level of human oversight. To bridge this gap and generate truly location-specific recommendations, future AI systems will need to integrate diverse data sources and variable information extraction approaches. For example, analyzing farmer data can result in identification of management practices and interactions that can increase crop yield and profit^20,21^ and can be a valuable data source. An approach that combines LLMs trained on scientific literature with the predictive power of machine learning models trained on randomized field trials, commercial performance data, and on-farm experimentation could provide more comprehensive and actionable results. Such a system would generate insights that are not only grounded in formal research documented in literature but are also refined by and validated against field-specific data, ultimately increasing both crop yield and profit at the field level.

The expert sentiment analysis further contextualizes these findings. While a strong majority of experts recognized the potential for AI to improve efficiency (77% agreement) and usefulness (58% agreement), there were significant reservations regarding trust and data sharing which aligns with farmers reluctance to share data^22^. Only 40% of experts expressed trust in AI, and nearly 19% were hesitant to share data. This ‘trust deficit’ is a major barrier, not only for the adoption of AI-generated advice but also for the development of more powerful, location-specific models. Overcoming this reluctance to share data is critical, as the commercial farm data currently held by farmers and their service providers is precisely the resource needed to train LLMs to provide tailored, field-specific recommendations. Future systems will need to create a value proposition where farmers see a direct benefit from contributing their data to a larger, anonymized pool that powers more accurate and personalized insights.

## Conclusions

Our findings show that LLMs, when guided by human experts, can serve as powerful tools to accelerate the synthesis of complex agricultural research. However, the generation of valuable farm management advice requires a deep understanding of context and practical application. The failure of a generalized plan to satisfy experts highlights that the future of agricultural AI lies in its ability to provide tailored, region or in most cases field-specific recommendations. Achieving this level of specificity will require moving beyond the synthesis of formal scientific literature to incorporate large-scale commercial performance data from on-farm settings. The success of these technologies will ultimately depend on building trust within the agricultural community. Future work should focus not only on improving the technical accuracy and specificity of AI models but also on creating transparent, secure, and collaborative systems that empower, rather than replace, human expertise. This study represents a crucial step in navigating the path toward an AI-assisted future for agriculture that balances technological potential with practical needs and inherent skepticism of its intended users.

## Supplementary Information

Below is the link to the electronic supplementary material.

Supplementary Material 1

## Data Availability

The data can be made available upon request. Contact corresponding author with any queries.

## Code availability

All LLM prompts used are provided in supplementary material.
