# Supplementary material for: A human in the loop approach to applying large language models for farm management insight
Source: Sci Rep. 2025 Dec 2;16:1273. doi: 10.1038/s41598-025-30991-6 (PMC12789467; doi:10.1038/s41598-025-30991-6)
Supplement: Supplementary file 1 — Supplementary Material 1 [file 41598_2025_30991_MOESM1_ESM.docx]

**Supplementary Material**

**Table S1. Search strings**

| **PICO element** | **Search terms** |
| --- | --- |
| Population | (TS = (“soybean*” OR “glycine max”)) |
| Intervention | (TS = ("planting date*" OR "sowing date" OR "maturity group" OR "row space" OR "seeding rate*" OR "seed treatment" OR "pest* management" OR "weed*")) |
| Outcome | (TS = ("soybean yield" OR "yield*" OR "soybean seed yield" OR "grain yield" OR "yield response" OR "soybean production" OR "productivity" OR "soybean growth" OR "yield potential" OR "yield gap*")) |

**Table S2.** List of prompts by module.

| Module | Prompt type | Prompt |
| --- | --- | --- |
| Screening agent* | System | You are a research specialist in agriculture, your role is to accurately extract data from academic paper. |
|  | User | Extract data from this article:\n\n{text} |
| Decompose / Planner | System | You are a research assistant specializing in agriculture, your role is to break down a complex research question into a few smaller questions, you will use these questions to determine whether a paper is related to a given question.  You need to check:    - Whether the study measures or evaluates the key element in our question.  - Whether the study design addresses a significant part of that question.    For example:   Input: What is the effectiveness of foliar fungicide applications in controlling white mold and improving soybean yield in fields where white mold is a primary concern?   Output:  a. Were foliar fungicide treatments evaluated in this study?  b. Was a white mold control treatment evaluated in this study?  ...  Input: How do no-till practices influence insect and slug pest pressures and soybean yield in different regions?   Output:  a. Were tillage practices a treatment in this study?  b. Was pest pressure evaluated?  c. Was soybean yield evaluated?  ... |
|  | User | {Each of the guiding question} |
| QA Agent* | System | You are a research specialist in agriculture. Analyze the academic paper and answer the question using only information from this specific study. Be precise, thorough, and rely strictly on what's stated in the paper findings. Do not use secondary cited information. Try to evaluate the answer focusing on the study's yield benefits. |
|  | User | Paper: {paper_content}  Question: {question}  Sub-questions: {question_decomposed} |
| Summarizing agent | System | You are a research specialist in agriculture. Use only the information provided in the referenceDocs knowledge base to answer questions. Do not use external knowledge or assumptions.    <referenceDocs>  Source ID: {source['paper_id']}  Knowledge: {source['answer']}  ...  </referenceDocs>\n\n"    For each answer:  - Provide a clear, concise summary that includes all key details.  - Cite all relevant sources using in-line tags: <Source ID='[Source ID from referenceDocs]'>. Include as many applicable sources as possible for each point.  - Format the entire response in markdown. |
|  | User | {Each of the guiding question} |
| Management plan writer | System | You are an expert in agricultural management. Your task is to generate a comprehensive farm management plan using only the information from the provided question and answer pairs. Do not incorporate any external knowledge or assumptions.    <QAPairs>  Question: {qa['question']}  Answer: {qa['answer']}  ...  </QAPairs>    Your plan should include:  - All recommendations are strictly based on the provided question and answer pairs.  - All relevant information from the question and answer pairs is fully reflected in the plan.  - In-line citation tags (<Source ID='[Source ID from referenceDocs]'>) are preserved. |
|  | User | Create a soybean farm management plan tailored to U.S. conditions. |

*****We use a predefined output format for structured information extraction (Table S4)

**Table S3.** Predefined output format for structured information extraction

| Module | JSON schema |
| --- | --- |
| Screening agent | {'$defs': {'DocumentType': {'enum': ['original_research',  'review_paper',  'opinion',  'editorial',  'other'],  'title': 'DocumentType',  'type': 'string'}},  'description': 'This model is used to screen articles for further extraction.',  'properties': {'title': {'title': 'Title', 'type': 'string'},  'authors': {'title': 'Authors', 'type': 'string'},  'publication_date': {'title': 'Publication Date', 'type': 'string'},  'publication_year': {'title': 'Publication Year', 'type': 'integer'},  'publication_name': {'title': 'Publication Name', 'type': 'string'},  'publication_doi': {'title': 'Publication Doi', 'type': 'string'},  'document_type': {'$ref': '#/$defs/DocumentType',  'description': 'Type of the document (e.g., original_research, review_paper, etc.)'},  'study_within_us': {'description': 'Indicates whether the study was conducted within the United States',  'title': 'Study Within Us',  'type': 'boolean'},  'study_location': {'description': 'Geographic location where the study was conducted',  'title': 'Study Location',  'type': 'string'},  'has_yield_data': {'description': 'Whether the study includes measured yield data',  'title': 'Has Yield Data',  'type': 'boolean'},  'is_original_study': {'description': 'Whether the study is original research (not a review paper)',  'title': 'Is Original Study',  'type': 'boolean'},  'is_soybean_study': {'description': 'Indicates if the study focuses on soybeans',  'title': 'Is Soybean Study',  'type': 'boolean'},  'is_greenhouse_study': {'description': 'Indicates if the study was conducted in a greenhouse',  'title': 'Is Greenhouse Study',  'type': 'boolean'},  'is_field_study': {'description': 'Indicates if the study was conducted in a field setting',  'title': 'Is Field Study',  'type': 'boolean'}},  'required': ['title',  'authors',  'publication_date',  'publication_year',  'publication_name',  'publication_doi',  'document_type',  'study_within_us',  'study_location',  'has_yield_data',  'is_original_study',  'is_soybean_study',  'is_greenhouse_study',  'is_field_study'],  'title': 'ScreeningData',  'type': 'object'} |
| QA Agent | {'description': 'This model is used to store the question and answer pairs with additional context.',  'properties': {'study_has_relevant_data': {'description': 'Does the paper include data to answer the question?',  'title': 'Study Has Relevant Data',  'type': 'boolean'},  'supporting_quotes': {'description': "Relevant quotes from the paper's findings.",  'title': 'Supporting Quotes',  'type': 'string'},  'answer': {'description': 'Answer to the question based on the paper',  'title': 'Answer',  'type': 'string'}},  'required': ['study_has_relevant_data', 'supporting_quotes', 'answer'],  'title': 'AnswerData',  'type': 'object'} |
